# Le pilomatricome: une tumeur à connaître

**DOI:** 10.11604/pamj.2014.18.182.4861

**Published:** 2014-06-25

**Authors:** Nada El Moussaoui, Badredine Hassam

**Affiliations:** 1 Service de Dermatologie, CHU Ibn Sina, Université Med V, Souissi, Rabat, Maroc

**Keywords:** Pilomatricome, tumeur, histologie, pilomatricoma, tumor, histology

## Image en médecine

Le pilomatricome, anciennement appelé épithélioma calcifié de Malherbe, est une tumeur cutanée annexielle bénigne rare, développée à partir des cellules de la matrice pilaire. Le pilomatricome survient pendant les deux premières décennies de la vie et est habituellement localisée à la partie supérieure du corps. Dans sa forme typique, il se traduit cliniquement par un petit nodule sous-cutané, solitaire, asymptomatique parfois douloureux. La taille habituelle est inférieure à 3 cm. Cependant, quelques cas de pilomatricomes géants ont été rapportés. Des formes familiales associant de multiples pilomatricomes ainsi que des maladies de système notamment la dystrophie myotonique ont été décrites. Le diagnostic est évoqué cliniquement et nécessite une confirmation histologique. Le pronostic est généralement bon. La transformation carcinomateuse reste controversée. La guérison sans récidive est la règle après exérèse chirurgicale. Nous rapportons le cas de Mme L.A., 48 ans, sans antécédents pathologiques notables, qui consulte pour une tumeur de la joue droite, polylobée, dure, bien limitée, de 4cm de grand axe, avec une infiltration sous cutanée dépassant la tumeur visible. L'examen locorégional ne trouve pas d'adénopathie satellite palpable. L’étude histologique d'un fragment biopsique objective la présence de cellules très basophiles ainsi que de cellules momifiées acidophiles formant des nappes circonscrites et des massifs épithéliaux en faveur d'un pilomatricome. Une exérèse chirurgicale est réalisée. Aucune récidive n'est détectée après un recul de 12 mois.

**Figure 1:**
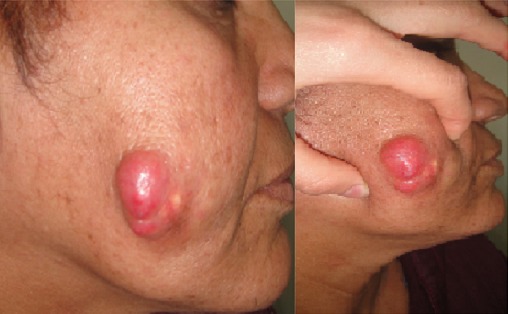
Tumeur dure polylobée de la joue gauche avec une infiltration sous cutanée

